# Supplementary material for: A model of dyadic merging interactions explains human drivers’ behavior from control inputs to decisions
Source: PNAS Nexus. 2024 Sep 24;3(10):pgae420. doi: 10.1093/pnasnexus/pgae420 (PMC11443968; doi:10.1093/pnasnexus/pgae420)
Supplement: pgae420_Supplementary_Data [file pgae420_supplementary_data.pdf]

A model of dyadic merging interactions explains human drivers' behavior from control inputs to decisions

Supplementary material

Olger Siebinga, Arkady Zgonnikov, and David Abbink

September 2024

This document contains additional information on the results presented in the paper "A model of dyadic merging interactions explains human drivers' behavior from control inputs to decisions." It contains additional results, details on the statistics, and a discussion on the model components and incentive functions.

1 Additional Results

1.1 Collisions

A limited number of trials ended in a collision. The model collided 29 times in 990 trials, and the human participants collided 28 times in 990 trials. Figure S1 shows the high-level outcome of all trials for the model and human behavior, including these collisions. Collisions happened infrequently and in all conditions in both the human and the model trials.

To gain more insight into why and how these collisions happened, we manually reviewed all collisions. Based on the human data, we identified 3 common categories of collisions (Table S1). Most collisions happened because both divers took the same action at the tunnel exit (i.e., they both sped up or slowed down). In many cases, one of the drivers tried to correct this mistake later on by changing their strategy. However, this intervention came too late, and the interaction ended in a collision. Most collisions in the model simulations also fall in this category. Furthermore, the model shows similar corrections in behavior as observed in the human data. See Figure S2 for a representative example of this type of collision for both the human and model data.

In other cases, neither of the drivers took action at the tunnel exit (Figure S3). As with the situations where the drivers initially took the same action, one of the drivers often tried to prevent the collision toward the end of the interaction. This type of collision happened more often in the human data than in the model simulations. But qualitatively, the type of collision is represented by the model.

In some collisions, one of the drivers took no action at the tunnel exit while the other did something but not enough to prevent a collision (Figure S4). As with the collisions where no driver took action, this type of collision happened more often in the human data than in the model simulations. Qualitatively, the behavior of the drivers leading up to the collision is represented by the model.

Finally, there are four human trials that ended in a collision but that don't fall in any of these categories (Table S1). In two cases, both drivers initially took opposing actions. This should lead

Table S1: A categorization of all collisions in the human data and model simulations. The table reports "pair-trial" combinations per category. The bottom row shows the total number of occurrences in each category.

Drivers take the same action		Drivers both do nothing		One driver does nothing, the other too little		Other	
human	model	human	model	human	model	human	model
1-32	1-41	1-43	2-101	1-27	3-23	1-2	
1-68	1-81	7-60	7-26	2-75	6-87	5-1	
1-86	1-109	8-39		3-18		5-4	
2-4	3-15	8-69		5-46		7-38	
2-9	4-102	8-110		8-24			
2-13	5-85						
2-16	5-108						
2-17	6-12						
2-18	6-35						
3-20	6-48						
5-6	6-58						
8-46	6-72						
9-32	6-106						
9-64	7-0						
	7-100						
	7-102						
	8-3						
	8-40						
	8-50						
	8-102						
	9-17						
	9-48						
	9-67						
	9-84						
	9-89						
14	25	5	2	5	2	4	0

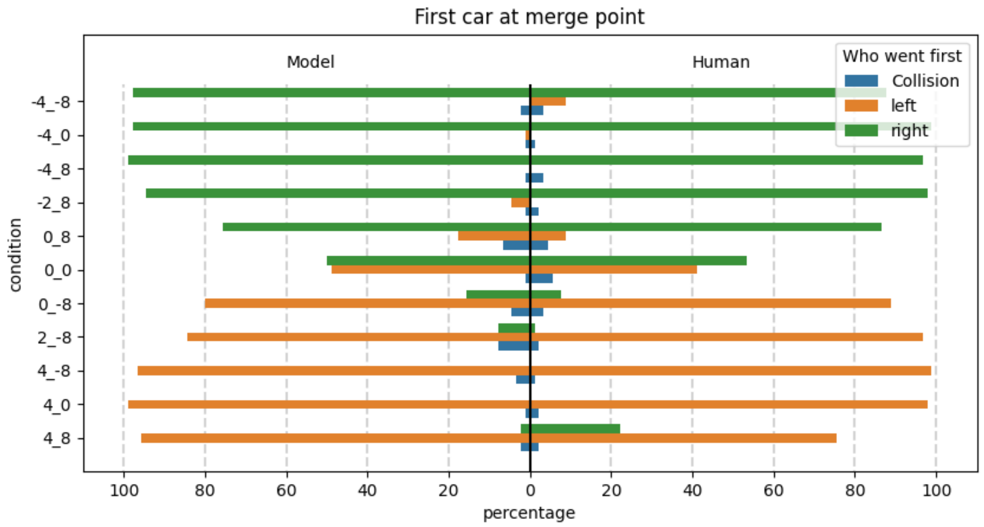

Figure S1: Which vehicle went first in which condition, including collisions

to a safe outcome. However, in these two trials, both drivers suddenly and simultaneously changed their strategy after some time. This is the same phenomenon described in the main paper as the example with ”miscommunication”; only these two times did it lead to a collision. This strategy change meant they changed the high-level outcome they were aiming for (i.e., the other driver would now go first). However, because it happened after some time, in these cases, there was not enough time left to reach this other solution safely. In the other two cases, the vehicles collided during the car-following phase near the end of the track. These phenomena were not replicated by the model. For the ”miscommunication” example in the paper, the model showed similar behavior but managed to prevent a collision by coming to a complete standstill.

Although this analysis of the trials ending in a collision is purely based on a manual qualitative evaluation, the results suggest that the model can represent the three most common phenomena that lead to a collision. The ratio at which these phenomena appear in the simulation does not completely correspond to the human data; however, with the low number of collisions, no definitive conclusion can be drawn about this ratio.

1.2 Conflict Resolution Time

To investigate the time it takes drivers to resolve this conflict, we use the Conflict Resolution Time (CRT) [1]. This metric describes the time between the start of the interaction (i.e., the tunnel exit) and the moment the drivers are no longer on a collision course. For details on how to calculate the CRT, and for an extended analysis of human behavior, see [2].

In human merging, the CRT depends not only on the kinematic conditions but also on the high-level outcome of a trial [2]. This can also be seen in Figure S5-A, where the CRTs for pair three show outliers in some conditions. These outliers represent the conditions where the other vehicle went first.

To account for this, we view the relationship between the kinematic conditions and CRT from the (kinematic) perspective of the vehicle that merged first (Figure S5-B). A positive number for

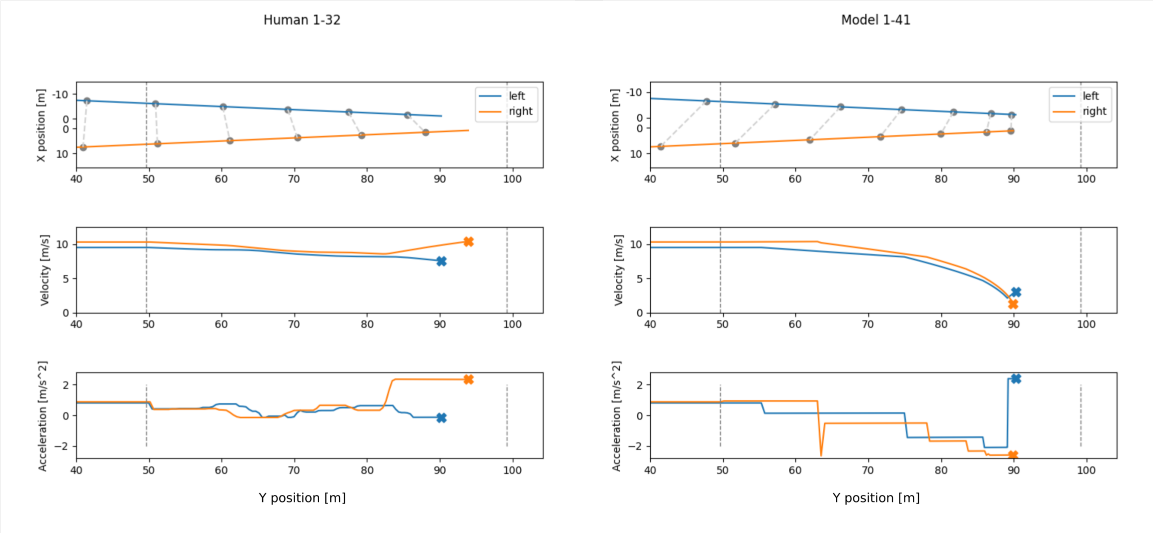

Figure S2: Two representative examples of an interaction that ended in a collision because both drivers took the same action when exiting the tunnel. In both the human data and the model simulation, both drivers decelerated when exiting the tunnel (first vertical dashed line). Although the amount of deceleration in the example of the human data is lower then in that of the model simulation. In both cases, one of the drivers attempted to prevent a collision by slightly accelerating toward the end of the interaction. However, this came too late to prevent a collision.

projected headway or relative velocity indicates an advantage for the vehicle that merged first. The effects of kinematics are small but significant and similar for the model and human data (Table S4). The largest difference is the velocity effect, which is twice as large in human behavior than in the model simulations. Overall, the model shows higher values for CRT than the human participants (Figure S5-C). There is a correlation between the model CRTs and the human CRTs.

Figure S5-A reveals that, particularly in conditions 4_8 and -4_-8, the model can not reproduce the CRTs for all pairs accurately. This could be related to the fact that in human behavior, these conditions show a substantial number of trials where the vehicle with the disadvantage merges first, while the model does not replicate this phenomenon (Figure S1. This could indicate that humans use a proxy to estimate the relative velocities and projected headways. However, the precise underlying mechanisms are unknown.

Table S2: Linear mixed-effects regression models describing the effect of projected headway, relative velocity, and the interaction all from the perspective of the vehicle that merged first on the CRT for the human (H) and model (M) (number of observations H/M: 962/961,log-likelihood H/M: -1038.5/-1319.9. Collisions were excluded.)

(a) Fixed effects

	Estimate	SE	Z	P-value	CI 0.025	CI 0.975
Intercept - H	1.61	0.11	15.18	4.9×10^{-10}	1.4	1.8
Intercept - M	1.84	0.10	17.65	9.5×10^{-70}	1.64	2.05
projected headway - H	-0.24	0.02	-15.3	2.1×10^{-47}	-0.28	-0.21
projected headway - M	-0.19	0.02	-10.7	1.3×10^{-26}	-0.22	-0.15
relative velocity - H	0.40	0.08	5.0	6.1×10^{-7}	0.25	0.56
relative velocity - M	0.23	0.206	0.09	6.8×10^{-3}	0.06	0.40
headway : relative velocity - H	-0.13	0.02	-6.08	1.7×10^{-9}	-0.18	-0.09
headway : relative velocity - M	-0.19	0.03	-6.96	3.5×10^{-12}	-0.24	-0.13

(b) Random effects

Pair	1	2	3	4	5	6	7	8	9
Random Intercept - H	-0.04	-0.35	-0.34	-0.037	-0.02	0.09	0.47	0.28	-0.04
Random Intercept - M	0.23	-0.32	-0.38	0.12	-0.09	0.06	0.31	0.21	-0.13

Table S3: Ordinary least squares linear regression on the model behavior as a function of human behavior in terms of CRT. Number of observations: 99, degrees of freedom residuals: 97, R-squared: 0.357, adjusted R-squared: 0.351

	Estimate	SE	t	P-value	Confidence interval 0.025	Confidence interval 0.975
Intercept	0.75	0.099	7.60	1.8×10^{-11}	0.56	0.95
Human input	0.65	0.09	7.35	6.4×10^{-11}	0.48	0.82

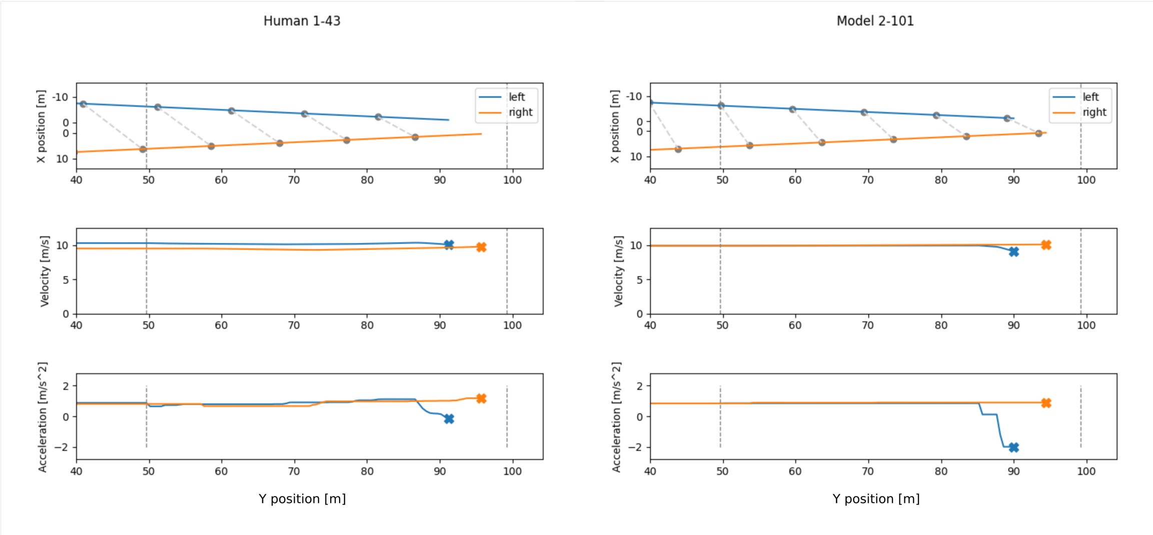

Figure S3: Two representative examples of an interaction that ended in a collision because both drivers took no action when exiting the tunnel. Toward the end of the interaction, the blue driver tried to prevent a collision by slowing down in both the human data and the model simulation.

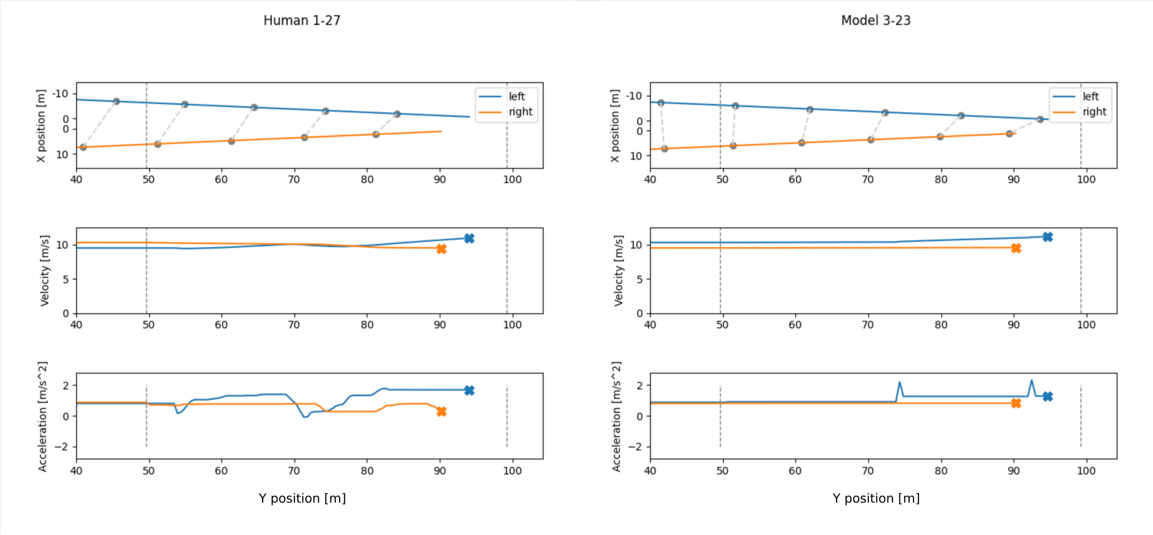

Figure S4: Two representative examples of an interaction that ended in a collision because the orange driver took no action when exiting the tunnel. This is best visible in the acceleration plot. The blue driver tried to prevent a collision by slightly accelerating after some time in both the human data and the model simulation. However, this wasn't enough to prevent a collision.

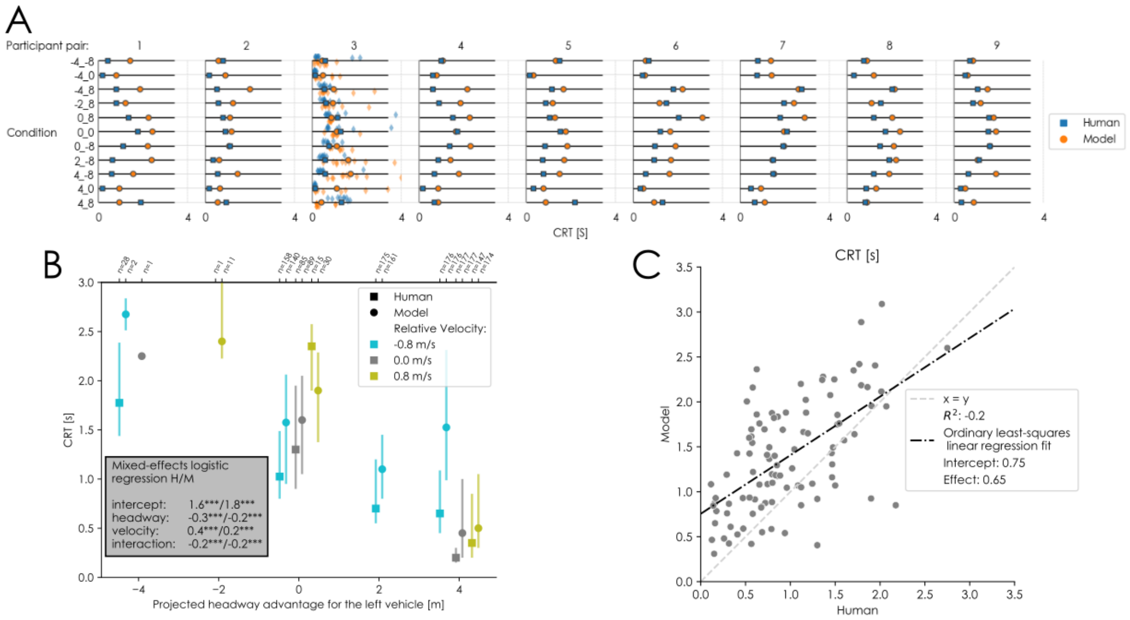

Figure S5: An overview of the behavior in terms of Conflict Resolution Time (CRT). **A**: the mean CRT values per pair, per condition. All underlying data of pair 3 is shown. **B**: the effects of projected headway and relative velocity on the CRT. The projected headway and relative velocity in this plot are seen from the perspective of the first merging vehicle. A positive number indicates an advantage for the vehicle that merged first. Markers show the mean values, lines indicate the interquartile ranges. **C**: the correlation between the model and human CRTs

2 Details on Statistics

We used multiple statistical models (mixed-effects regression models and linear regression models) to compare the model's behavior to human behavior in the results section of the paper. The details of all statistical models are presented here per level of behavior (i.e., figure in the paper).

2.1 High-level decisions

To investigate the effects of the kinematic conditions on the high-level outcome of the experiment, we fitted a logistic regression model (Table S4) to the proportion of the trials where the left vehicle merges first: $p \sim \Delta v + \Delta a$, where p is the probability of left merging first and Δv and Δx are the relative velocity and projected headway respectively. A random intercept per pair was included in the model. Collisions were excluded from this analysis.

To investigate the correlation between the model's output and human behavior, we fitted an ordinary least-squares linear regression to the mean deviation from the initial velocity per participant per high-level outcome (e.g., the left driver's behavior in all trials where the right driver merged first). The details of this regression can be found in Table S5.

2.2 Safety margins

To investigate the effects of the kinematic conditions on the gap drivers keep at the merge point, we fitted a linear mixed-effects regression model (Table S6) to the gap: $g \sim |\Delta v| + |\Delta x| + \Delta v * \Delta x$, where g is the gap and Δv and Δx are the relative velocity and projected headway respectively. A random intercept per pair was included in the model.

To investigate the correlation between the model's output and human behavior, we fitted an ordinary least-squares linear regression to the mean gap per pair per condition. The details of this regression can be found in Table S7.

2.3 Control inputs

To investigate the effects of the kinematic conditions on the absolute maximum deviation from the initial velocity, we fitted a linear mixed-effects regression model (Table S8) to the absolute deviation of the initial velocity: $a \sim \Delta v + \Delta x + \Delta v * \Delta x$, where a is the maximum absolute deviation from the initial velocity and Δv and Δx are the relative velocity and projected headway respectively. Collisions were excluded from this analysis and a random intercept per driver was included in the model.

To investigate the correlation between the model and human input behavior, we fitted an ordinary least-squares linear regression to the mean maximum absolute deviation from the initial velocity per driver per condition. The details of this regression can be found in Table S9.

3 Model Components

The model we present in the paper is based on the communication-enabled interaction framework. This framework states that a model of traffic interactions can be composed of four base components: plan, belief, communication, and risk. Removing one of these components will break the model. Without a planning module, the modeled drivers will not plan anything and, thus, not move. Without the belief, they will not consider each other and thus crash. Without communication, the belief cannot be updated, and the drivers will not respond dynamically to the other's actions (i.e., the

Table S4: Mixed-effects logistic regression models describing the effect of projected headway and relative velocity on which driver merged first for the human (H) and model (M) (number of observations H/M: 962/961,log-likelihood H/M: -191.0/-190.4. The parameters for the human data were previously published [2]. Collisions were excluded, the left vehicle going first was labeled as 1, right first as 0.

(a) Fixed effects

	Estimate	SE	Z	P-value	Confidence interval 0.025	0.975
Intercept - H	-0.32	0.212	-1.50	1.3×10^{-1}	-0.73	0.10
Intercept - M	-0.03	0.345	-0.07	9.4×10^{-1}	-0.70	0.65
Projected headway - H	1.15	0.080	14.4	7.0×10^{-47}	0.99	1.31
Projected headway - M	1.35	0.104	12.9	6.2×10^{-38}	1.14	1.55
Relative velocity - H	-3.413	0.321	-10.6	2.9×10^{-26}	-4.04	-2.78
Relative velocity - M	-1.752	0.226	-7.77	7.9×10^{-15}	-2.19	-1.31

(b) Random effects

Pair	1	2	3	4	5	6	7	8	9
Random Intercept - H	-0.54	-0.42	-1.17	0.06	-0.13	-0.51	0.16	-0.22	-0.13
Random Intercept - M	-0.41	1.06	-0.94	-0.52	-0.99	0.19	1.77	-0.53	0.13

Table S5: Ordinary least squares linear regression on the model behavior as a function of human behavior in terms of individual contribution per high-level outcome. Number of observations: 36, degrees of freedom residuals: 34, R-squared: 0.903, adjusted R-squared: 0.900

	Estimate	SE	t	P-value	Confidence interval 0.025	0.975
Intercept	-0.09	0.08	-1.09	2.8×10^{-1}	-0.254	0.08
Human input	1.04	0.06	17.76	8.9×10^{-19}	0.924	1.16

Table S6: Linear mixed-effects regression models describing the effect of absolute headway, absolute relative velocity, and the interaction of signed headway and relative velocity on the gap that drivers keep between the vehicles at the merge point for the human (H) and model (M) (number of observations H/M: 962/961,log-likelihood H/M: -1990.9/-2167.1. Collisions were excluded.)

(a) Fixed effects

					CI	
	Estimate	SE	Z	P-value	0.025	0.975
Intercept - H	4.13	0.45	9.14	6.1×10^{-20}	3.24	5.01
Intercept - M	5.21	0.28	18.43	7.7×10^{-76}	4.66	5.77
Absolute projected headway - H	0.15	0.04	4.23	2.4×10^{-5}	0.08	0.22
Absolute projected headway - M	-0.21	0.043	-4.95	7.5×10^{-7}	-0.30	-0.13
Absolute relative velocity - H	-0.18	0.17	-1.1	0.28	-0.52	0.15
Absolute relative velocity - M	0.065	0.206	0.32	0.75	-0.34	0.47
Headway : relative velocity - H	0.18	0.03	6.04	1.5×10^{-9}	0.12	0.24
Headway : relative velocity - M	0.21	0.036	5.89	4.0×10^{-9}	0.14	0.29

(b) Random effects

Pair	1	2	3	4	5	6	7	8	9
Random Intercept - H	-0.49	1.21	1.94	-0.01	-0.35	1.07	-1.61	-1.86	0.09
Random Intercept - M	-0.15	0.91	0.89	0.25	0.08	-0.27	-0.97	-0.16	-0.57

Table S7: Ordinary least squares linear regression on the model's gap-keeping behavior as a function of human gap-keeping behavior. Number of observations: 99, degrees of freedom residuals: 97, R-squared: 0.178, adjusted R-squared: 0.169

					Confidence interval	
	Estimate	SE	t	P-value	0.025	0.975
Intercept	3.2	0.34	9.5	1.7×10^{-15}	2.53	3.87
Human gap	0.34	0.07	4.58	1.4×10^{-5}	0.190	0.48

Table S8: Linear mixed-effects regression models describing the effect of project headway, relative velocity, and the interaction of projected headway and relative velocity on the absolute maximum deviation from the initial velocity that drivers use for the human (H) and model (M) (number of observations H/M: 1980/1980,log-likelihood H/M: -2482/-3850)

(a) Fixed effects

	Estimate	SE	Z	P-value	CI 0.025	CI 0.975
Intercept - H	1.93	0.16	12.2	2.1×10^{-34}	1.62	2.24
Intercept - M	2.14	0.19	110.09	1.4×10^{-28}	1.76	2.51
Projected headway - H	-0.23	0.02	-12.35	4.8×10^{-35}	-0.27	-0.19
Projected headway - M	-0.23	0.038	-6.14	8.4×10^{-10}	-0.31	-0.16
Relative velocity - H	-0.55	0.09	-5.99	2.2×10^{-9}	-0.73	-0.37
Relative velocity - M	0.55	0.18	2.98	2.9×10^{-3}	0.18	0.91
Headway : relative velocity - H	0.22	0.03	7.56	8.7×10^{-14}	0.16	0.27
Headway : relative velocity - M	-0.07	0.058	1.25	0.21	-0.19	0.04

(b) Random effects

Pair	1		2		3		4		5	
Driver	left	right	left	right	left	right	left	right	left	right
Random intercept - H	0.11	-0.29	-0.53	0.89	1.41	-0.82	-0.27	0.25	0.63	-0.39
Random intercept - M	0.50	-0.20	-1.13	0.84	0.82	-1.09	0.23	0.14	0.50	-0.73
Pair	6		7		8		9			
Driver	left	right	left	right	left	right	left	right		
Random intercept - H	0.53	-0.15	-0.98	0.21	0.18	-0.65	0.10	-0.23		
Random intercept - M	0.05	0.40	-0.75	0.32	0.57	-0.27	-0.00	-0.19		

Table S9: Ordinary least squares linear regression on the model's absolute maximum deviation from the initial velocity as a function of human maximum deviations. Number of observations: 297, degrees of freedom residuals: 295, R-squared: 0.505, adjusted R-squared: 0.504

	Estimate	SE	t	P-value	Confidence interval 0.025	Confidence interval 0.975
Intercept	0.61	0.10	5.9	1.0×10^{-8}	0.406	0.812
Human velocity deviation	0.90	0.05	17.4	5.1×10^{-47}	0.796	0.999

outcome will only depend on the initial state). And finally, without risk perception, the drivers will never update their plan and thus be unresponsive. However, one part of the model could be removed without breaking the system altogether (i.e., it is optional). This is the concept of the incentive functions we introduced in this paper.

To investigate the impact of the incentive functions on the model's behavior, we ran all simulations with the same parameters but with the incentive functions disabled. This practically means that the modeled drivers use their base values for the upper and lower risk thresholds as fixed values (Table 3 in the main paper). The dynamic incentive functions (equations 10 and 11 and Table 2 in the main paper) are not used.

Disabling the incentive functions has no substantial quantitative effect for most of the analyses we performed in the main paper (Firgure S6). The largest difference between the models with and without the incentive functions can be seen in the CRT comparison. The effect of the linear regression between the model with incentive and human data is stronger than for the model without incentive functions. The incentive functions have little effect on how much action the drivers take, but they have an effect on the timing.

This can also be seen qualitatively (Figure S7) in some pairs in certain conditions. Since the incentive functions are linear combinations of the headway and velocity difference, their effect is larger in conditions where these differences are larger. Figure S7 shows the velocity traces for condition $4_- - 8$, with the traces of pair 3 highlighted. It shows that the incentive functions have an effect on (mostly the left driver's) timing. The left driver acts early in the interactions with the incentive functions, while without the incentive functions, they act later.

Although the effects (and thus arguably the added benefits) of using the incentive functions are minor, we decided to use them in the model because they play a major role in the fitting procedure. We fit the model based on the grid search data using a linear mixed-effects model. The results of this fitting procedure (Table 2 in the main paper) show a statistically significant effect ($p < 0.05$) on the risk thresholds for all parameters except for the velocity difference effect on the lower threshold ($p = 0.51$). Combined with the influence on the quantitative results shown here, this is sufficient motivation to include the incentive functions in the model.

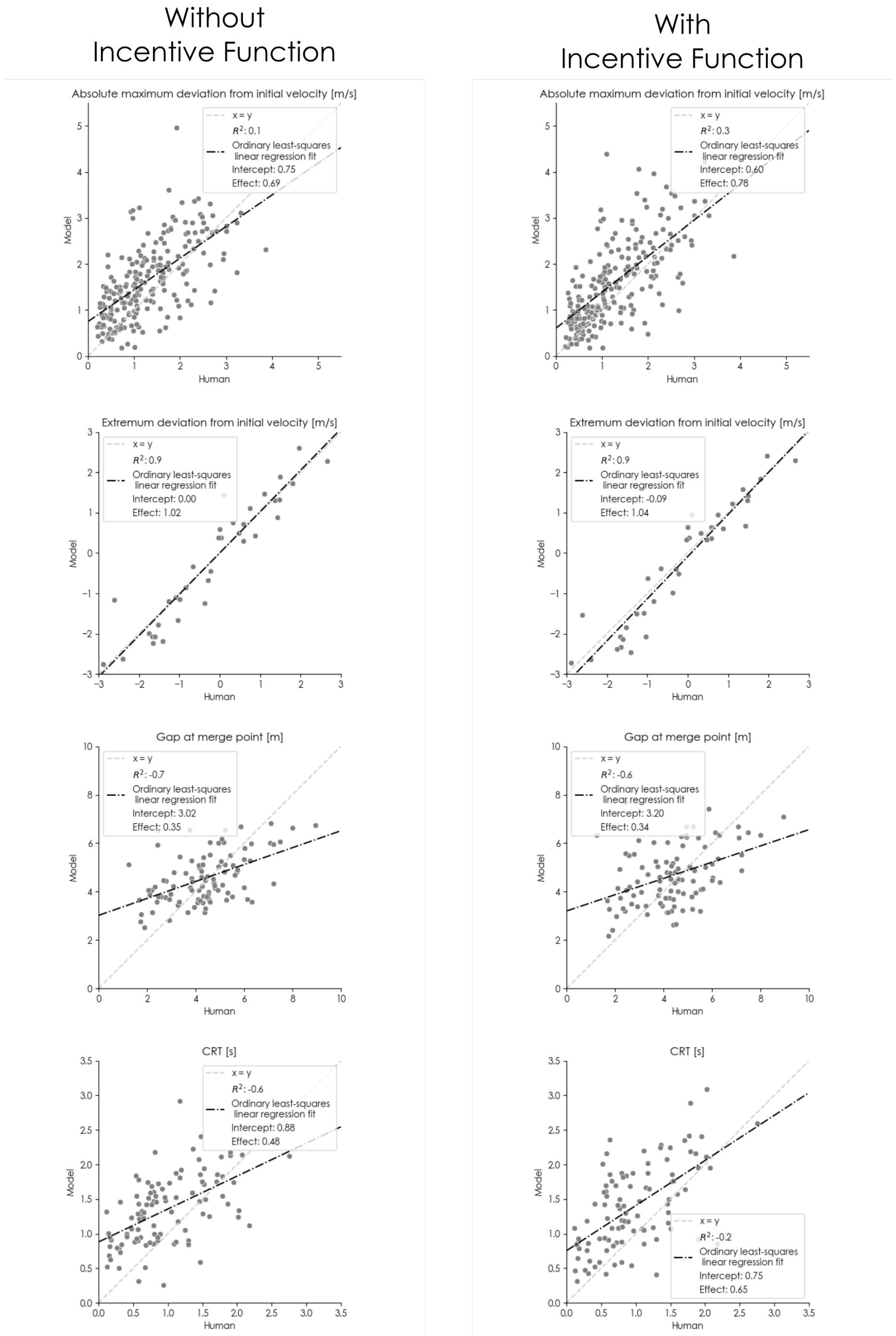

Figure S6: Comparison of the quantitative results between the model with incentive functions (as presented in the main paper) and without incentive functions.

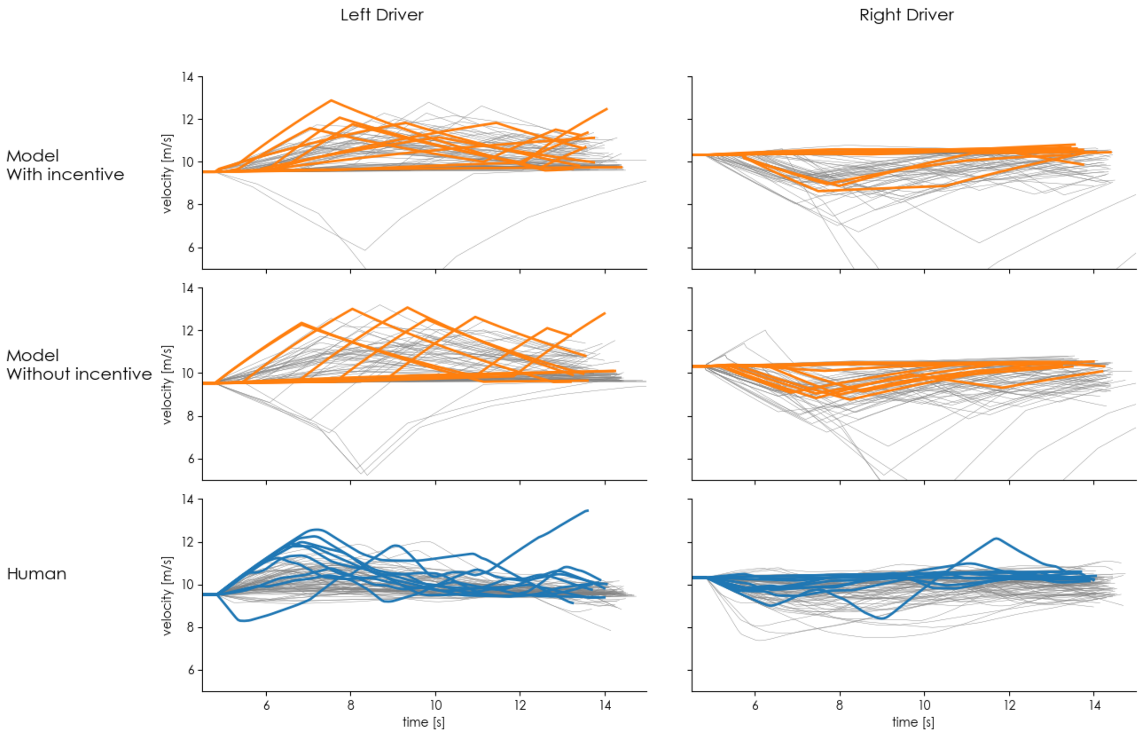

Figure S7: All velocity traces for conditions $4_- - 8$ for the human drivers, the original model (as presented in the paper), and the model without the incentive functions. The highlighted trajectories are those of participant pair 3.

References

[1] Olger Siebinga, Arkady Zgonnikov, and David Abbink. Interactive merging behavior in a coupled driving simulator: Experimental framework and case study. Human Factors in Transportation, 60:516–525, 2022.

[2] Olger Siebinga, Arkady Zgonnikov, and David A. Abbink. Human Merging Behavior in a Coupled Driving Simulator: How Do We Resolve Conflicts? IEEE Open Journal of Intelligent Transportation Systems, 5:103–114, 2024.
